# Chiral Luminophore Guided Self-Assembly of Achiral Block Copolymers for the Amplification of Circularly Polarized Luminescence

**DOI:** 10.1021/acsmacrolett.4c00188

**Published:** 2024-05-30

**Authors:** Sheng-Wei Shao, Puhup Puneet, Ming-Chia Li, Tomoyuki Ikai, Eiji Yashima, Rong-Ming Ho

**Affiliations:** † Department of Chemical Engineering, National Tsing Hua University No. 101, Section 2, Kuang-Fu Road, Hsinchu 30013, Taiwan, R.O.C.; ‡ Department of Biological Science and Technology, Center for Intelligent Drug Systems and Smart Bio-devices (IDS2B), National Yang Ming Chiao Tung University, Hsinchu 300, Taiwan, R.O.C.; § Department of Molecular and Macromolecular Chemistry, Graduate School of Engineering, Nagoya University, Chikusa-ku, Nagoya, Aichi 464-8603, Japan

## Abstract

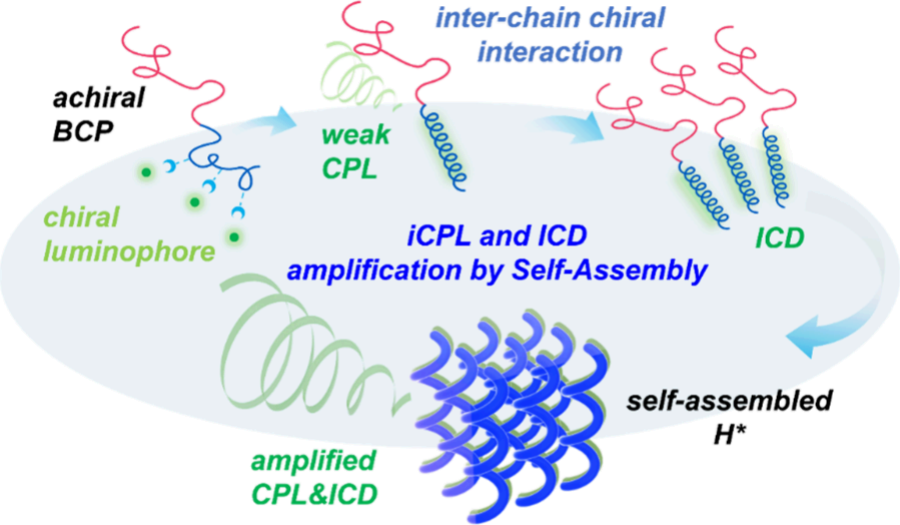

This work aims to examine the effect of self-assembly on the chiroptic responses of the achiral block copolymer (BCP) polystyrene-*b*-poly(ethylene oxide) (PS-*b*-PEO) associated with chiral luminophores, (*R*)- or (*S*)-1,1′-bi-2-naphthol ((*R*)- or (*S*)-BINOL), through hydrogen bonding. With the formation of a well-ordered helical phase (H*), significantly induced circular dichroism (ICD) signals for the PEO block in the mixture can be found. Most interestingly, a remarkable amplification with an extremely large dissymmetry factor of luminescence (*g*_lum_) from 10^–3^ to 0.3 (i.e., induced circular polarized luminescence (iCPL) behavior) for the chiral BINOLs in the mixture can be achieved by the formation of the helical phase (H*) via mesochiral self-assembly. As a result, by taking advantage of BCP for mesochiral self-assembly, it is feasible to create a nanostructured monolith with substantial optical activities, offering promising applications in the design of chiroptic devices.

Circularly polarized luminescence (CPL) materials recently have drawn intensive attention due to its promising applications in 3D displays,^1^ data storage,^2^ chiroptical sensors,^3^ and organic light-emitting diodes.^4,5^ In addition to the physical method involving a linear polarizer and quarter-wave plates, circularly polarized light can be directly generated from chiral luminescent materials, thereby preventing energy loss during the transition between the plates. The CPL activity is generally observed in π-conjugated chiral small molecules,^6,7^ polymers,^8^ and lanthanide complexes.^9^ Note that most of the chiral organic luminophores exhibit a moderate signal of CPL, with a luminescence dissymmetry factor (*g*_lum_) in the range of 10^–5^∼10^–3^, which may not serve the purpose for engineering applications. Self-assembly has been a popular candidate that offers a possibility for precisely controlling the arrangement of luminophores and enhancing specific properties. Recent remarkable studies have explored strategies to amplify the CPL activity, such as self-assembly of luminophores with hierarchical textures,^10−14^ doping of achiral dye into chiral nematic liquid crystals,^15^ coassembly of π-conjugated polymers with helicenes,^16^ and unique luminescence from upconversion^17^ and the aggregation-induced emission (AIE) emitters.^18^ An intriguing recent approach for fabricating CPL-active materials through the self-assembly pathway involves associating the guest luminophore with an assembled chiral host, referred to as “chiral host–achiral luminescent guest”, and has been widely demonstrated.^13^ However, only a few reports exist regarding an “achiral host–chiral luminescent guest”. Moreover, very few of them are capable of fabricating well-ordered films for practical applications.

Block copolymers (BCPs) are well-known for their ability to self-assemble into well-ordered phases via microphase separation of distinct covalently jointed blocks.^19−21^ Moreover, they are recognized for their capability to produce precisely ordered films. Conventionally, BCPs can self-assemble into well-ordered microstructures such as sphere (S), cylinder (HC), double gyroid (DG), and lamellae (L) phases. Interestingly, a peculiar helical phase (H*) was found in the self-assembly of poly(lactide)-based chiral block copolymers (BCP*)^22,23^ via chirality transfer at different length scales with homochiral evolution^24^ that was further evidenced by using poly(cyclohexylglycolide) (PCG)-based BCPs* for self-assembly,^25^ suggesting the generalization of self-assembled behavior of BCP*. Yashima and co-workers have demonstrated the feasibility to induce chirality of achiral polyacetylenes via noncovalent interaction with chiral dopants, giving single-handed helicity, commonly referred to as ICD behavior.^26,27^ Watkins and co-workers further demonstrated the feasibility to introduce chirality into BCPs for mesochiral self-assembly of PEO-*b*-PtBA by association with chiral tartaric acid.^28,29^

Herein, this work aims to demonstrate the feasibility for amplification of the CPL activity of chiral luminophore from mesochiral self-assembly of BCP. As illustrated in Figure 1a, the association of achiral BCP and chiral luminophore can be achieved by a host–guest interaction. With the induced chirality for the achiral BCP by the chiral luminophore, a helical polymer with exclusive helicity for an associated constituted block in the BCP can be formed via an intrachain chiral interaction, giving the formation of BCP* (Figure 1b); this behavior is referred to as the ICD behavior of achiral BCP. By taking advantage of mesochiral self-assembly, microphase separation with interchain chiral interaction (Figure 1c) gives rise to the formation of H* through an induced twisting and shifting mechanism. This process amplifies the CPL activity for the chiral luminophores (Figure 1d), referred to as the induced CPL (iCPL) behavior of the chiral luminophore. A representative system, polystyrene-*b*-poly(ethylene oxide) (PS-*b*-PEO) as the achiral host associated with chiral quest, (*R*)- or (*S*)-1,1′-bi-2-naphthol ((*R*)- or (*S*)-BINOL) for the host–guest interaction through hydrogen bonding (Figure 1e), is used for the demonstration of the suggested approach to the aimed ICD and iCPL behaviors. As found in this study, it is possible to create the self-assembled mixture in the thin-film state as optical film with significant ICD behavior for the achiral PEO in the mixture and extraordinary amplification of iCPL for *g*_lum_ from the chiral BINOL up to ∼100-fold (*g*_lum_ = ± 0.3). These enhancements are attributed to the arrangement of helical chain packing from the mesochiral self-assembly with H* formation at which a significant interchain chiral interaction can be achieved by the twisting and shifting of a microphase-separated domain for ordering. The observed amplification not only contributes to the improved self-assembly, but also facilitates the transfer of chirality, progressing from molecular scale to chain conformation and further extending to the mesoscale level. This comprehensive transformation thus leads to an overall enhancement in the ICD of the achiral BCP host and the iCPL of the chiral luminophore guest.

**Figure 1:**
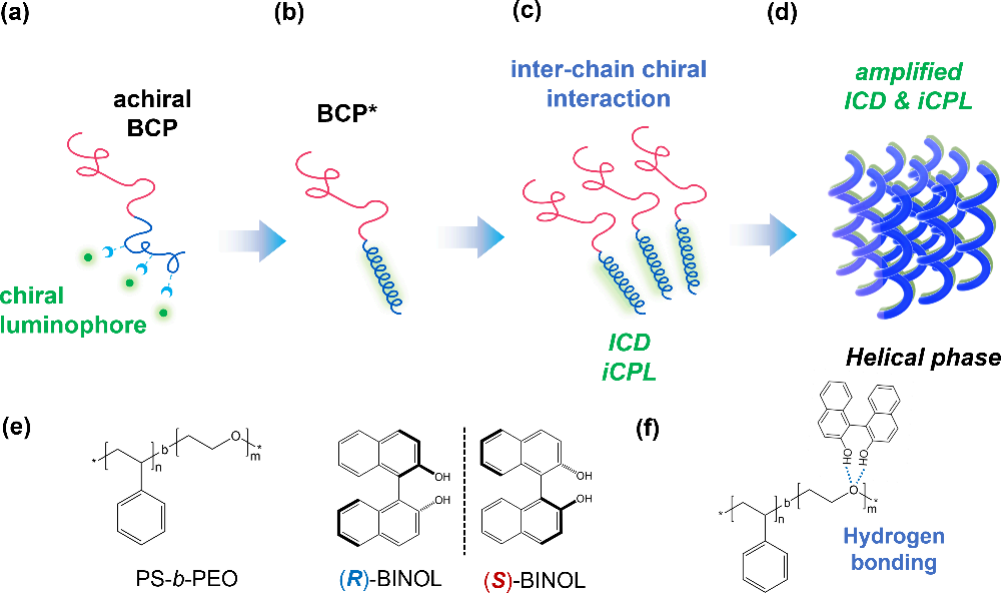
Schematic illustration of (a) association of achiral BCP with a chiral luminophore via hydrogen bonding; (b) Formation of BCP* after the host–guest association with intrachain chiral interaction; (c) Interchain chiral interaction, giving rise to weak ICD and iCPL; (d) Amplification of the ICD and iCPL resulting from the formation of H*; (e) Representative “achiral host and chiral guest” system with achiral PS-*b*-PEO and chiral BINOL; (f) Intermolecular hydrogen bonds between PS-*b*-PEO and chiral BINOL.

To achieve the aimed ICD and iCPL behaviors for achiral PS-*b*-PEO and chiral BINOL, respectively, it is necessary to create the host–guest interaction in which hydrogen bonding is expected via the association of PEO and BINOL. As shown in Figure S1a, ^1^H NMR spectra show a downfield-shifted broad resonance peak from 5.05 to 5.35 ppm of moderately acidic −OH protons in (*R*)-BINOL due to the association with a lone-pair of electrons located at the oxygen linkage of the PEO and a slight upfield shift of CH_2_ protons of the PEO from 3.62 to 3.61 ppm due to the formation of a chelating complex via hydrogen bonding (Figure S1c), suggesting the formation of the aimed association. Consistently, similar results can be found in the mixture of PS-*b*-PEO and (*S*)-BINOL (Figure S1b,d). In contrast to the ^1^H NMR spectra from the PEO, the NMR peak of the PS block remains unchanged with either (*R*)- or (*S*)-BINOL (Figure S1e), suggesting that there is no association between the PS and the chiral BINOL. Those results indicate that the association of the chiral BINOL is selective with the PEO block. With the selective association, it is expected to give the formation of BCP* with the formation of a static helical chain for the PEO due to the intrachain chiral interaction; similar behaviors have been found in the mixtures of BCP and chiral dopants for the ICD behavior.^28,29^

Figure S2a shows the ECD spectra of (*R*)- and (*S*)-BINOL in solution with characteristic multiple bisignate Cotton bands originating from the π–π* transition of ^1^B_b_ couplings of axially twisted two naphthalene units of the intrinsic chiral BINOLs. In the presence of PS-*b*-PEO, ECD signals remain unchanged in solution, suggesting that hydrogen bonding would not affect the axial chirality of BINOLs. As a result, it is intuitive to suggest that the forming BCP* after the host–guest association adopts the chirality from the chiral BINOL that might give rise to the helical PEO chain with exclusive helicity due to the intrachain chiral interaction. Figure S2b shows the VCD spectra of (*R*)- and (*S*)-BINOL in solution with a Cotton band and bisignate Cotton band at 1125 and 1145 cm^–1^, resulting from the C–O stretching of axially twisted two naphthalenol of the chiral BINOLs. In the presence of PS-*b*-PEO, the broad peak of the C–O–C vibration of the PEO at 1094 cm^–1^ can be identified, and the VCD signals of BINOL remain unchanged in solution (Figure S2c), reflecting that the hydrogen bonding would not affect the axial chirality of BINOLs. Note that there is no discernible ICD behavior of the C–O–C vibration at around 1094 cm^–1^, which might be attributed to the flexibility and nature of the PEO backbone in the solution state; consequently, even with the association of chiral BINOL, there is no obvious ICD signal being recognized.

To further examine the ICD behavior, the VCD experiments in the solid state were conducted in comparison with the results from solution. Note that, in contrast to the results from solution, there is a slight increase in the absorption for the samples in the solid state; yet, VCD spectra give rise to recognized signals with bisignate Cotton bands of C–O–C vibration modes at 1084 and 1093 cm^–1^ resulting from the ICD behavior (Figure S2d). The spectra with a split-type Cotton effect are attributed to an interchain chiral interaction; note that the vibration of C–O–C is parallel to the main chain, thus, giving the ICD behavior from the aggregation of the BCP* chains in the solid state. Accordingly, in the solid state, one can expect the formation of a static helical PEO chain conformation resulting from an intrachain chiral interaction due to the association of chiral BINOL with a larger helical inversion barrier because of the closer distance between polymer chains. The appearance of the ICD signals suggests the aimed interchain chiral interaction was driven from the formation of a static helical PEO chain induced by the association of the chiral BINOL. As a result, those results ensure the suggested approach to induced chirality from the host–guest interaction.

To explore the ICD behavior stemming from interchain chiral interaction, the ordering process from the mesochiral self-assembly was carried out by solvent annealing. As shown in Figure 2a, the TEM image of PS-*b*-PEO displays dark PEO cylinders within a bright PS matrix, arranged as a hexagonally packed lattice, as depicted in the inset, for the neat PS-*b*-PEO sample from solvent annealing. Notably, this mass contrast is achieved through preferential staining of RuO_4_ with the PEO block. The formation of the hexagonally packed cylinder phase (HC) can be further confirmed by one-dimensional (1D) SAXS measurements (Figure 2d), revealing reflections occurring at the relative *q* values of . Upon interaction with chiral BINOL, in contrast to the neat PS-*b*-PEO, there is an obvious variation on the morphological evolution after solvent annealing; as shown in Figures 2b and 1c, the mixtures of PS-*b*-PEO/(*R*)- and (*S*)-BINOL_0.2_ both reveal dark PEO helices within the bright PS matrix. Corresponding 1D SAXS results (Figure 2d) with reflections at relative *q* values of  further confirm that the forming helices assemble into a hexagonal lattice. The emergence of H* indicates that the preferential association of chiral BINOL to the PEO block in the PS-*b*-PEO indeed triggers a twisting and shifting of the PEO microdomain,^23,30^ giving the phase transition from HC to H*. The forming H* is further evidence that the observed ICD behavior of the PS-*b*-PEO is driven by the association of the chiral BINOL via an interchain chiral interaction. Owing to the ordering process, it is reasonable to expect the amplification of the interchain chiral interaction.

**Figure 2:**
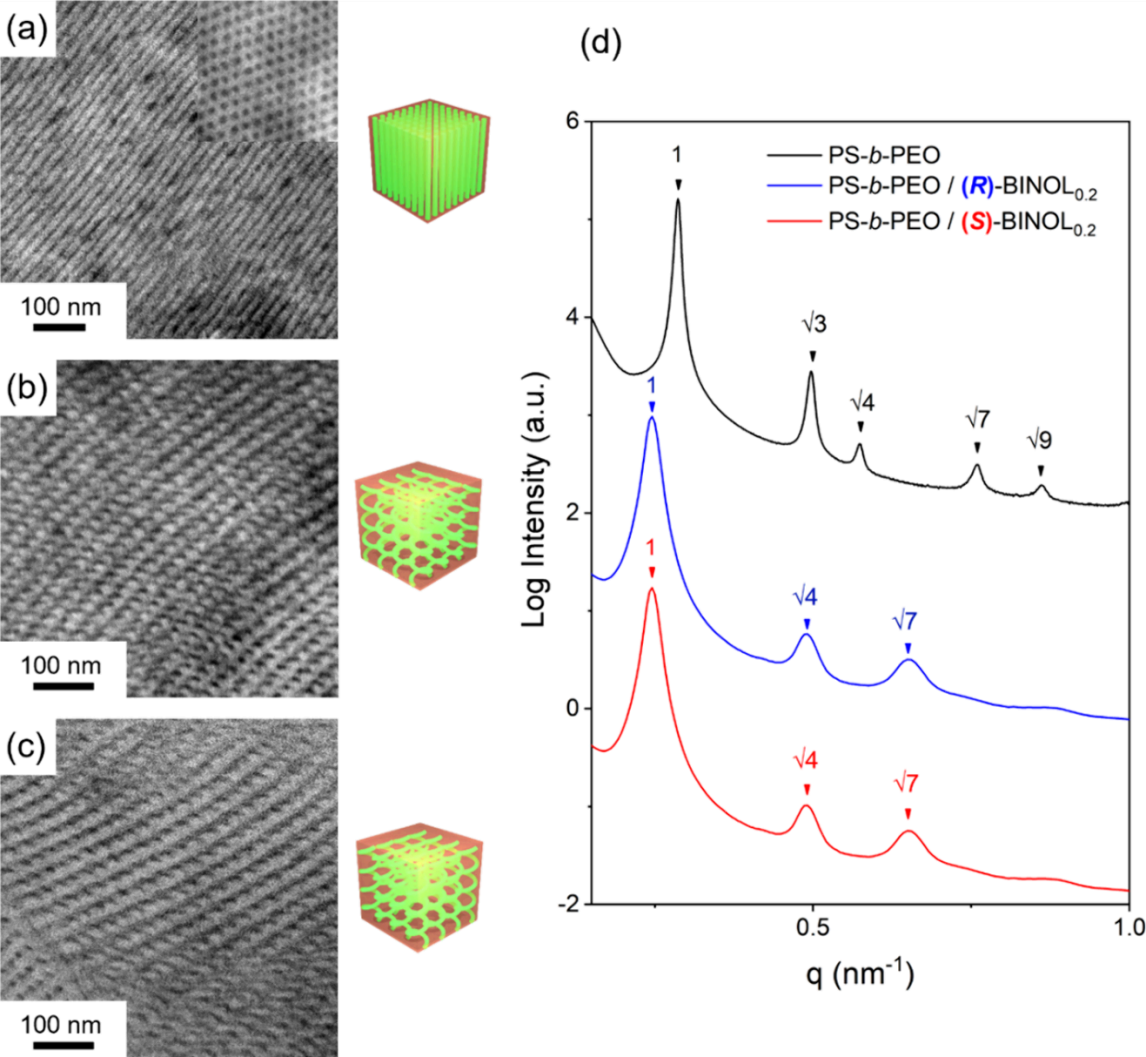
TEM micrographs of the (a) HC phase from self-assembled PS-*b*-PEO (inset shows hexagonal packing of cylinders); (b) H* phase from self-assembled PS-*b*-PEO/(*S*)-BINOL_0.2_ and (c) PS-*b*-PEO/(*R*)-BINOL_0.2_; (d) Corresponding 1D SAXS profiles.

To examine the effects of microphase separation and the corresponding ordering with the formation of H*, VCD experiments were traced on the ordering process. Remarkably, upon self-assembly through solvent annealing to achieve a high degree of ordering, a significant enhancement of the ICD of mirror-imaged split-type Cotton bands for the C–O–C vibrations emerges in both PS-*b*-PEO/(*R*)- and (*S*)-BINOL_0.2_. This amplified ICD is obviously attributed to the formation of an enhanced interchain chiral interaction. The corresponding IR spectra give rise to the intensification of the C–O–C vibration peak at 1090 cm^–1^, which is further evidence of the enhancement of dipole moments from the self-assembling process. More importantly, with the association of chiral BINOLs, as shown in Figure 3, significant enhancement of the ICD signals can be found at the C–O–C vibration of PEO after the formation of H*. As a result, the chirality transfer from the molecular chirality results from the ordering process for the formation of H* with homochiral evolution via interchain chiral interaction.

**Figure 3:**
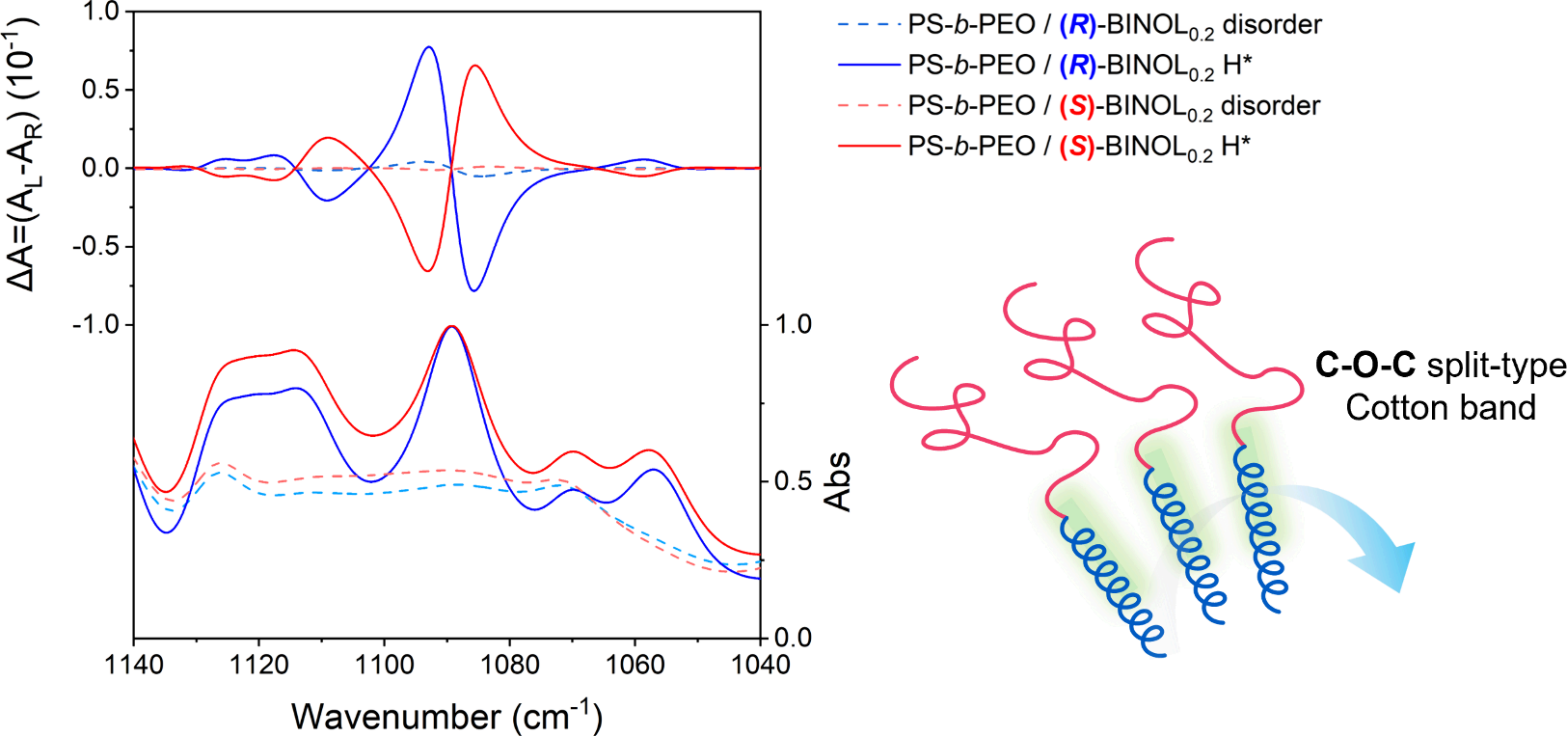
FTIR and corresponding VCD spectra of chiral BINOLs to the conformational chirality of PEO are effectively leveraged to give intensified ICD signals disordered and ordered in the thin-film state of PS-*b*-PEO/(*R*)-BINOL_0.2_ and PS-*b*-PEO/(*S*)-BINOL_0.2_.

Given the significant enhancement of the ICD signals for the PEO block in BCP*, attributed to an association with chiral BINOL, the formation of the static helical PEO chain suggests that BINOL would be associated with the PEO in a preferred helical sense. Figure S4a shows the CPL/PL spectra of isolated BINOLs and PS-*b*-PEO/(*R*)- and (*S*)-BINOL_0.2_ in a dilute solution (10^–4^ M in THF); no significant alteration in chiroptical activities can be observed since there is no substantial interchain chiral interaction from the PEO chains in solution, even with the association, in line with the previous solution state ECD and VCD results. In contrast to the inherent BINOL in the solid state (*g*_lum_ = 0.002), a moderately enhanced CPL activity (*g*_lum_ = 0.009) becomes apparent in the solid-state PS-*b*-PEO/(*R*)- and (*S*)-BINOL_0.2_ within the disordered phase (i.e., the cast thin film before solvent annealing for ordering (Figure S4b). The marginal increase in chiroptical activities implies that the presence of randomly oriented helical chains in the disordered state gives rise to the slight amplification effect. Remarkably, as shown in Figure 4a, the formation of H* leads to an ultra-amplification of optical activity, with the *g*_lum_ value (0.3) exceeding that of the intrinsic chiral BINOL by more than 2 orders of magnitude. Notably, this gives rise to mirror-image bisignate signals for PS-*b*-PEO/(*R*)- and (S)-BINOL_0.2_ at 363 nm, indicating a blue shift compared with the disordered phase signal (373 nm). The extraordinary enhancement of CPL activity is attributed to the hierarchical arrangement of luminophores via interchain chiral interaction within the self-assembled helical microdomains, possessing a preferred handedness in the multiscale. Consequently, the helically aligned electric transition moments (normal to magnetic transition moments) for π–π* transition vectors culminate in the ultimate enhancement of the differential transition probability (*W*_gn_),^31^ which directly correlates with CPL activity. Figure 4b systematically contrasts the *g*_lum_ values of chiral BINOL at different states. In contrast to the negligible CPL signals from intrinsic chiral BINOLs, a discernible amplification of CPL activity is evident with the formation of the H* phase, as illustrated in Figure 1d, while the enhancement in CPL signals from the disordered phase remains noticeable but insignificant. These outcomes unequivocally demonstrate that the ultra-amplification of CPL arises during the self-assembly of achiral PS-*b*-PEO in the presence of BINOL, facilitated by the creation of a well-ordered helical microdomain as the H* phase, leading to intensified interchain chiral interaction (i.e., mesochiral self-assembly). To further explore the influence of hydrogen bonding between BINOL and PEO on the ICD and iCPL behaviors, methoxy and dimethoxy derivatives of BINOLs, denoted as mBINOL (with one OH group) and dmBINOL (without OH group), respectively, were introduced for comparison of BINOL with two OH groups (Figure S5; for the CD and CPL spectra of (*R*)- and (*S*)-mBINOL, -dmBINOL, and their mixtures with PS-*b*-PEO in a dilute solution, see Figures S6 and S7, respectively). As shown in Figure S8, in the case of mBINOL, the formation of hydrogen bonding between PEO and mBINOL might not be sufficiently robust to produce the desired iCPL, resulting in relatively weak iCPL signals. In the absence of an OH group (dmBINOL), precipitation will occur during the film preparation resulting from the aggregation of the dmBINOL due to the absence of hydrogen bonding association with PS-*b*-PEO (Figure S9). Therefore, the formation of a chelating complex through hydrogen bonding is crucial, particularly for the PEO system, which exhibits a flexible chain confirmation nature. Consequently, this process is essential for the creation of a stable helical polymer chain, ultimately leading to the desired iCPL and ICD behaviors via self-assembly.

**Figure 4:**
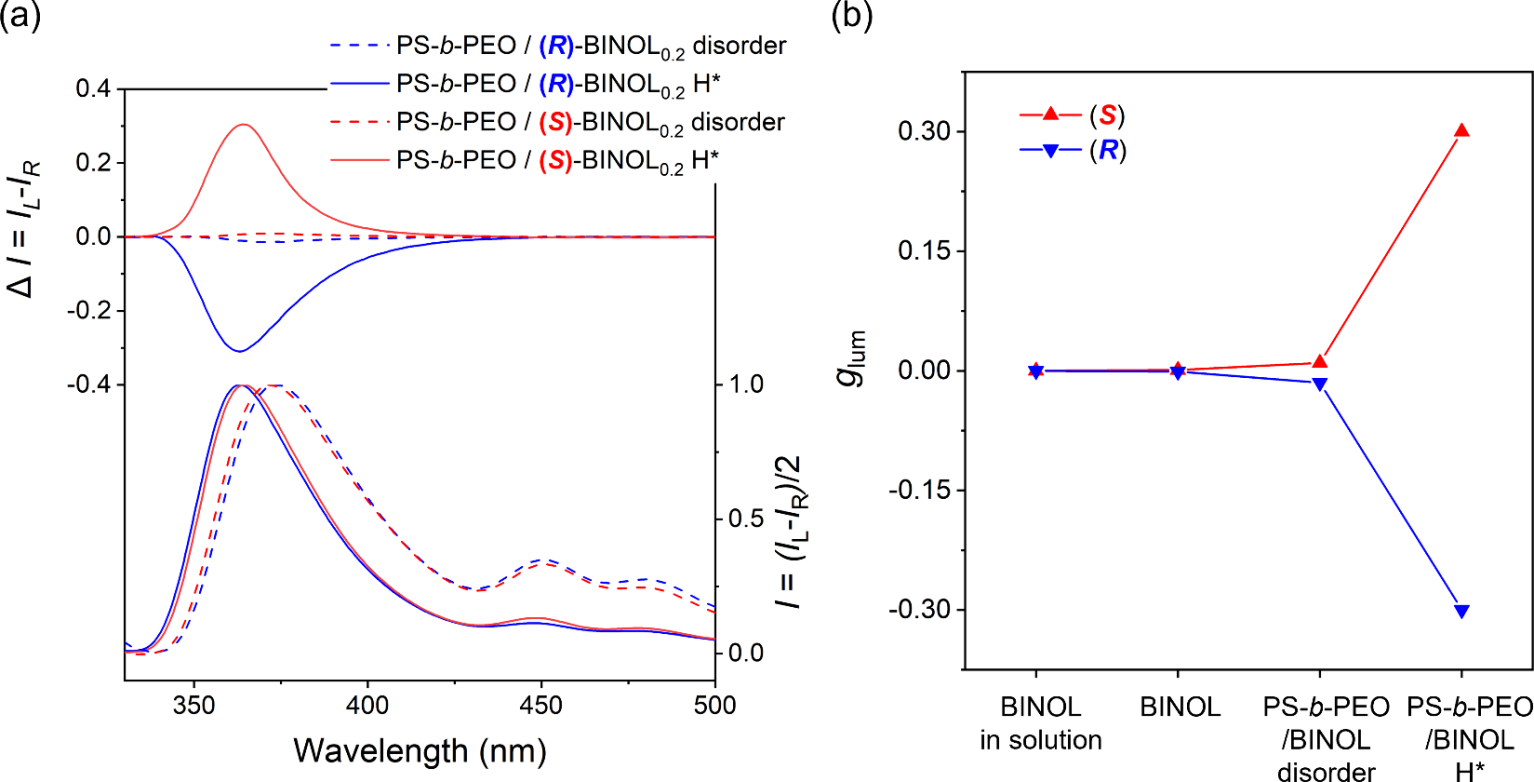
CPL and corresponding PL spectra of disordered and H* phases of (a) PS-*b*-PEO/(*R*)-BINOL_0.2_ and PS-*b*-PEO/(*S*)-BINOL_0.2_ in the solid state; (b) Comparison of *g*_lum_ of (*R*)- and (*S*)-BINOLs in solution and bulk and PS-*b*-PEO/(*R*)-BINOL_0.2_ and PS-*b*-PEO/(*S*)-BINOL_0.2_ with disordered and H* phases.

For a systematic study, mixtures with various doping ratios of PS-*b*-PEO and BINOL were prepared. Remarkably, with the incorporation of 0.1 equiv of (*R*)- or (*S*)-BINOL into PS-*b*-PEO, a peculiar self-assembled phase, DG, can be formed (Figure 5). Intriguingly, the calculated effective volume fraction was approximately 0.28, a value typically outside the window for DG. This finding aligns with our earlier discovery that an increase in twisting power results in the enlargement of DG windows, and further escalation of twisting power leads to the emergence of the H*.^32^ Note that a DG composed of a pair of continuous, interpenetrating but independent, coherent single gyroid (SG) networks at which one forms a clockwise network and the other forms an anticlockwise network, give the most thermodynamically stable phase.^33,34^ Specifically, it is an achiral phase from a mesochiral self-assembly. Contrastingly, as shown in Figure S10, the observed silence in the VCD signal, in comparison to the results obtained from the H* forming sample, suggests that the racemic mesophase has the effect of canceling out the ICD signal. Additionally, the significant reduction in CPL activity of the DG from the mixtures of PS-*b*-PEO/(*R*)- or (*S*)-BINOL_0.1_ (Figure S11) further reinforces this notion. The nullification of the CPL signals implies the presence of an equal population of left- and right-handed chiral networks, as illustrated in Figure 1d. This observation underscores the critical role of mesochiral self-assembly in influencing the observed ICD and iCPL behaviors.

**Figure 5:**
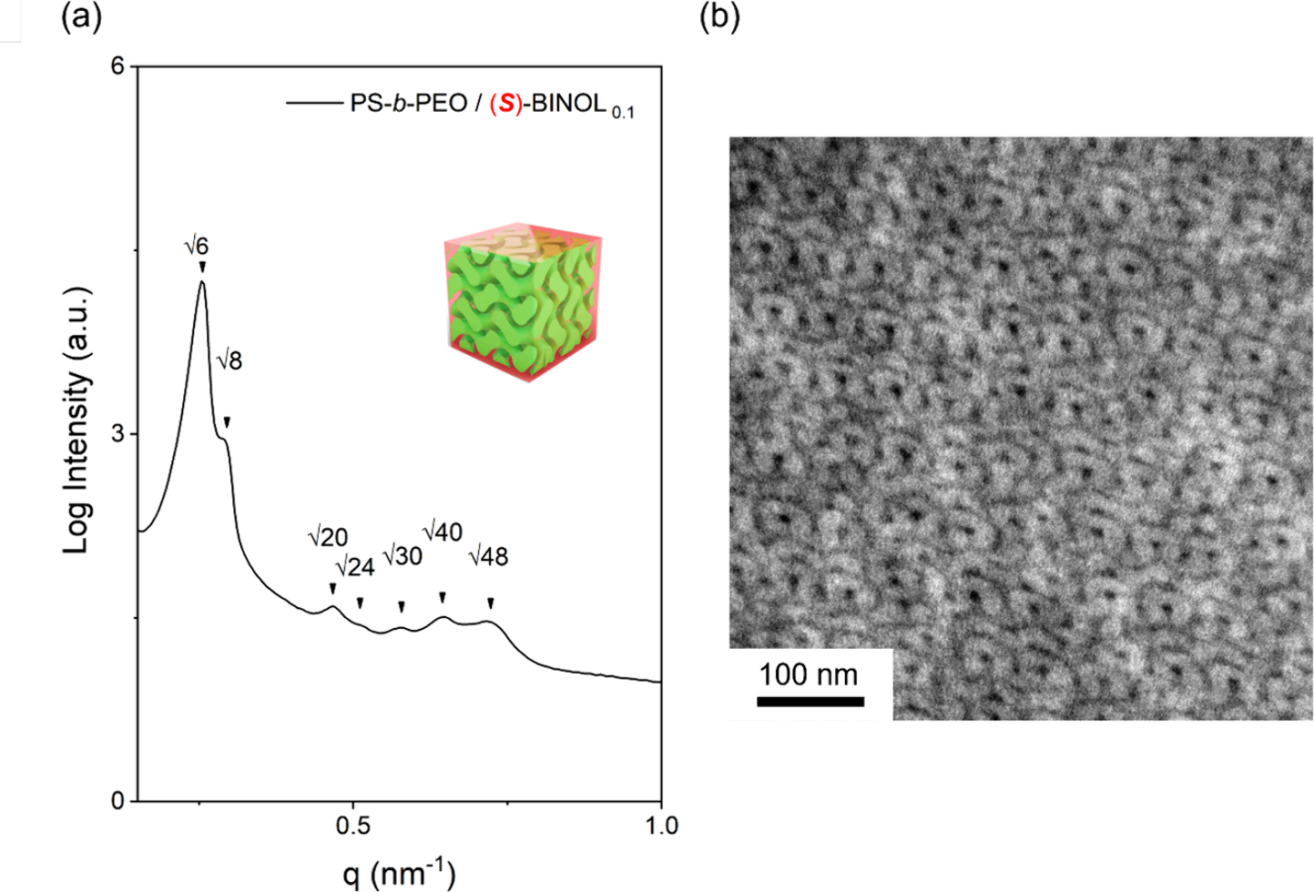
(a) 1D SAXS profile and (b) TEM projection image of the DG phase from the self-assembly of PS-*b*-PEO/(*S*)-BINOL_0.1_.

In conclusion, this work demonstrates a simple approach to induce chirality for achiral BCP through an association with chiral luminophores. With the effect of mesochiral self-assembly for ordering, ultra-amplification of the iCPL behavior could be achieved in the thin-film state after the formation of a well-ordered helical phase. The induced CPL activity of an extremely large *g*_lum_ reaching 0.3 could be mapped out through the hierarchical arrangement of luminophores in a one-handed H* phase, whereas the disordered phase and DG network phase behave as achiral entities, thus giving feeble *g*_lum_ in the range of 10^–3^. This conceptually intriguing and simple approach provides a pathway for the fabrication of a nanostructured monolith with strong CPL activity that will stimulate research exploration for chiroptical applications.

## Supporting Information Available

Sample preparation, experimental detail, and ^1^NMR, ECD, VCD, and CPL results (PDF)
