# Supplementary material for: Chiral Luminophore Guided Self-Assembly of Achiral Block Copolymers for the Amplification of Circularly Polarized Luminescence
Source: ACS Macro Lett. 2024 May 30;13(6):734–40. doi: 10.1021/acsmacrolett.4c00188 (PMC11191678; doi:10.1021/acsmacrolett.4c00188)
Supplement: Supplementary file 1 — mz4c00188_si_001.pdf [file mz4c00188_si_001.pdf]

Supporting Information

Chiral Luminophore Guided Self-Assembly of Achiral Block Copolymers for Amplification of Circularly Polarized Luminescence

Sheng-Wei Shao [a,€], Puhup Puneet [a,€], Ming-Chia Li[b], Tomoyuki Ikai[c], Eiji Yashima[c], Rong-Ming Ho[a,*]

[a] Department of Chemical Engineering, National Tsing Hua University No. 101, Section 2, Kuang-Fu Road, Hsinchu, Taiwan 30013, R.O.C.

[b] Department of Biological Science and Technology, Center for Intelligent Drug Systems and Smart Bio-devices (IDS2B), National Yang Ming Chiao Tung University, Hsinchu 300, Taiwan.

[c] Department of Molecular and Macromolecular Chemistry, Graduate School of Engineering, Nagoya University, Chikusa-ku, Nagoya, Aichi 464-8603, Japan.

€ Equal contribution

*Corresponding authors

Email: rmho@mx.nthu.edu.tw

1. Instrumentation

1.1 Spectroscopic Analysis by Vibrational Circular Dichroism (VCD)

The VCD spectra were measured on a JASCO FVS-6000 spectrometer in a cylindrical BaF_2 cuvette with a path length of 50 μm. The IR absorption and VCD spectra could be obtained simultaneously at a resolution of 4 cm^{-1} with 4500~6000 accumulations. The baseline was calibrated using (+)/(−)α-pinene as a standard.

1.2 Electronic Circular Dichroism Spectroscopy (ECD)

The ECD spectra were measured on JASCO J-815 spectrometer in a cylindrical quartz cuvette with light path-length of 1.0 mm for solution samples whereas solid-state samples were casted on the quartz plate. The UV-Vis absorption and CD spectra could be obtained simultaneously during measurements at scanning rate of 100 nm/min with 5~10 accumulations. The parameter of g_{abs} was experimentally determined using the expression $\Delta\varepsilon/\varepsilon$ = (ellipticity (in mdeg)/32980)/absorbance at the CD maxima. The dissymmetry factor of the circular polarization at the S_0 state (g_{abs}) was evaluated as $g_{abs} = (\varepsilon_L - \varepsilon_R)/(1/2(\varepsilon_L + \varepsilon_R))$, where ε_L and ε_R are the extinction coefficients for *l*- and *r*-CP light, respectively.

1.3 Circular Polarized Luminescence Spectroscopy (CPL)

The CPL spectra were measured on JASCO CPL-300 spectrometer in a cylindrical cuvette for solution samples with light path-length of 1.0 mm for solution samples whereas solid-state samples were casted on the quartz plate. The CPL and corresponding photo-luminescence spectra could be obtained simultaneously during measurements.

The dissymmetry factor of the circular polarization at the S_1 state (g_{lum}) was evaluated as $g_{lum} = (I_L - I_R)/(1/2(I_L + I_R))$ where I_L and I_R are the intensities of the signals for *l*- and *r*-CP light, respectively, under the incident UP light, similar to the parameter of g_{lum}, calculated as $\Delta I/I$ = (ellipticity (in mdeg)/(32980/ln10))/total PL intensity (in volts) at the CPL maxima.

1.4 Transmission electron microscopy (TEM)

The transmission electron microscopy (TEM) images were obtained using the

mass contrast with a JEOL JEM-2100 LaB_6 transmission electron microscope (at an accelerating voltage of 200 kV). The bulk samples were sectioned at room and cryo condition by Leica Ultra-microtome. Subsequently, the microsections were collected on gold grids (100 mesh). For PS-*b*-PEO sample, staining was accomplished by exposing the samples to the vapor of RuO_4 for 3 hours. The RuO_4 would interact with the lone pairs on the oxygen, rendering those microphase-separated domains dark in TEM *via* mass contrast.

1.5 Small-angle X-ray scattering (SAXS)

Small-angle X-ray scattering (SAXS) experiments were conducted at the synchrotron X-ray beam-line TLS 23A and TPS 13A at the National Synchrotron Radiation Research Center (NSRRC). SAXS data were collected with a 15-keV X-ray beam and a sample-to-detector distance of 10 m. One-dimensional (1D) linear profile was obtained by integration of the 2D pattern. The scattering intensity profiles were plotted with the scattering intensity (I) versus the scattering vector (q), where $q = 4\pi\lambda^{-1}\sin\theta$ defined by the scattering angle 2θ and X-ray wavelength λ (0.8266 Å used).

2. Experimental method

2.1 Materials

Polystyrene-block-polyethylene oxide (PS-*b*-PEO, Polymer Source), 1,1'-Bi-2-naphthol (BINOL, Alfa Aesar), THF (Sigma-Aldrich) were used as received without further purification,

2.2 Preparation of self-assembled PS-*b*-PEO blended with BINOLs

Self-assembled samples of PS-*b*-PEO blended with BINOL were prepared by solution casting from THF at room temperature. Samples were first dissolved in THF at a concentration of 10 wt% and then placed into glass bottles sealed with aluminum foils at room temperature. A hole (0.8 mm in diameter) was punched in the center of the aluminum foil to let the solvent evaporate. From the weight loss of the solution, the drying time and the amounts of the residual solvents can be estimated. The drying time was largely extended to avoid retaining solvent in the as-cast sample. The bulk sample further thermal annealing for 100 ^oC for 2 hours, and finally quenched to room temperature at 150 ^oC/min for further characterization.

2.3 Self-assembled morphology characterization

The transmission electron microscopy (TEM) images were obtained using the mass contrast with a JEOL JEM-2100 LaB_6 transmission electron microscope (at an accelerating voltage of 200 kV). The bulk samples were sectioned at room and cryo condition by Leica Ultra-microtome. Subsequently, the microsections were collected on gold grids (100 mesh). For PS-*b*-PEO sample, staining was accomplished by exposing the samples to the vapor of RuO_4 for 3 hours. The RuO_4 would interact with the lone pairs on the oxygen, rendering those microphase-separated domains dark in TEM *via* mass contrast. Small-angle X-ray scattering (SAXS) experiments were conducted at the synchrotron X-ray beam-line TLS 23A and TPS 13A at the National Synchrotron Radiation Research Center (NSRRC). SAXS data were collected with a 15-keV X-ray beam and a sample-to-detector distance of 10 m. One-dimensional (1D) linear profile was obtained by integration of the 2D pattern. For example, in **Figure 2(d)**, the PS-*b*-PEO/(S)-$BINOL_{0.2}$ data is integrated from **Figure S12**, and all samples exhibit no notable anisotropic effect. The scattering intensity profiles were plotted with the scattering intensity (I) versus the scattering vector (q), where $q = 4\pi\lambda^{-1}\sin\theta$ defined by the scattering angle 2θ and X-ray wavelength λ (0.8266 Å).

2.4 Measurements of CD, UV-Visible spectra, VCD, FTIR spectra and CPL spectra

The film samples were either drop-coated or dip-coated onto quartz substrates to create a uniform film thickness of approximately 4000 nm for subsequent measurements. Prior to acquiring the spectra, each sample underwent solvent annealing in a saturated tetrahydrofuran (THF) vapor environment for 2 hours, followed by allowing the solvent to evaporate over a period of about 2 days. Subsequently, the film sample underwent further thermal annealing at 100°C for 2 hours. CD and UV-Visible spectra were performed using a JASCO J-815 spectrometer at room temperature. Scanning parameters were set as scanning speed, 50 nm/min; digital integration time, 8s; three accumulations; data pitch, 0.1 nm; and bandwidth, 13 nm. The anisotropy g factor was calculated according to the following equation: g=CD/(32980×abs) where abs is the absorption. The VCD spectra were measured on a JASCO FVS-6000 spectrometer in a cylindrical BaF2 cuvette with a path length of 50 μm. The IR absorption and VCD spectra could be obtained simultaneously at a resolution of 4 cm^{-1} with 4500~6000 accumulations. The baseline was calibrated using (+)/(−)α-pinene as a standard. The CPL spectra were measured on JASCO CPL-300 spectrometer in a cylindrical cuvette for solution samples with light path-length of 1.0 mm for solution samples whereas solid-state samples were casted on the quartz plate. The CPL and corresponding photo-luminescence spectra could be obtained simultaneously during measurements. The dissymmetry factor of the circular polarization was evaluated as $g_{lum} = (I_L - I_R)/(1/2(I_L + I_R))$ where I_L and I_R are the intensities of the signals for *l*- and *r*-CP light, respectively, under the incident UP light, similar to the parameter of glum, calculated as ΔI/I = (ellipticity (in mdeg)/(32980/ln10))/total PL intensity (in volts) at the CPL maxima.

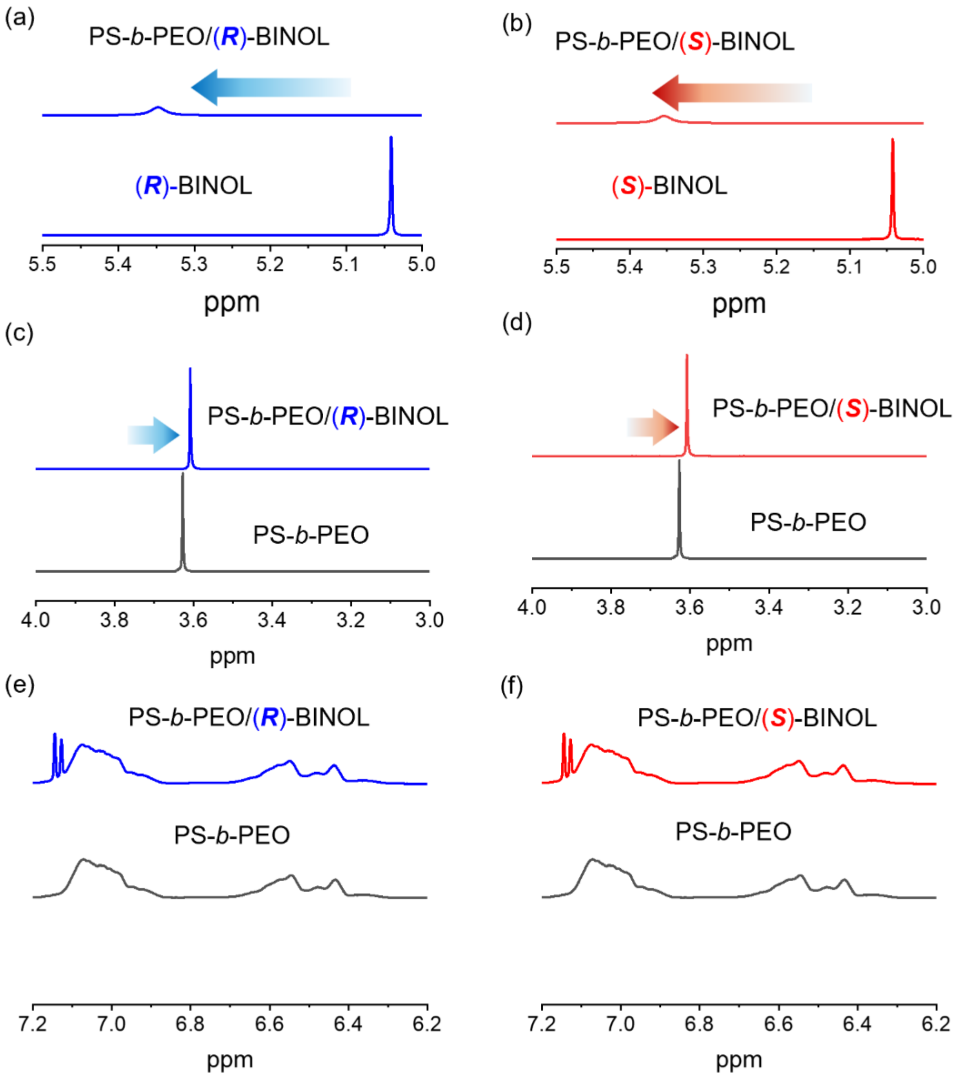

Figure S1. ^{1}H-NMR spectra of 4wt% (A) (*R*)-BINOL and PS-*b*-PEO/(*R*)-BINOL$_{0.2}$, and (B) (*S*)-BINOL and PS-*b*-PEO/(*S*)-BINOL$_{0.2}$ in $CDCl_3$ solution; (C) (*R*)-BINOL and PS-*b*-PEO/(*R*)-BINOL$_{0.2}$, and (D) (*S*)-BINOL and PS-*b*-PEO/(*S*)-BINOL$_{0.2}$ in $CDCl_3$ solution; (E) (*R*)-BINOL and PS-*b*-PEO/(*R*)-BINOL$_{0.2}$ and (F) (*S*)-BINOL and PS-*b*-PEO/(*S*)-BINOL$_{0.2}$ in $CDCl_3$ solution.

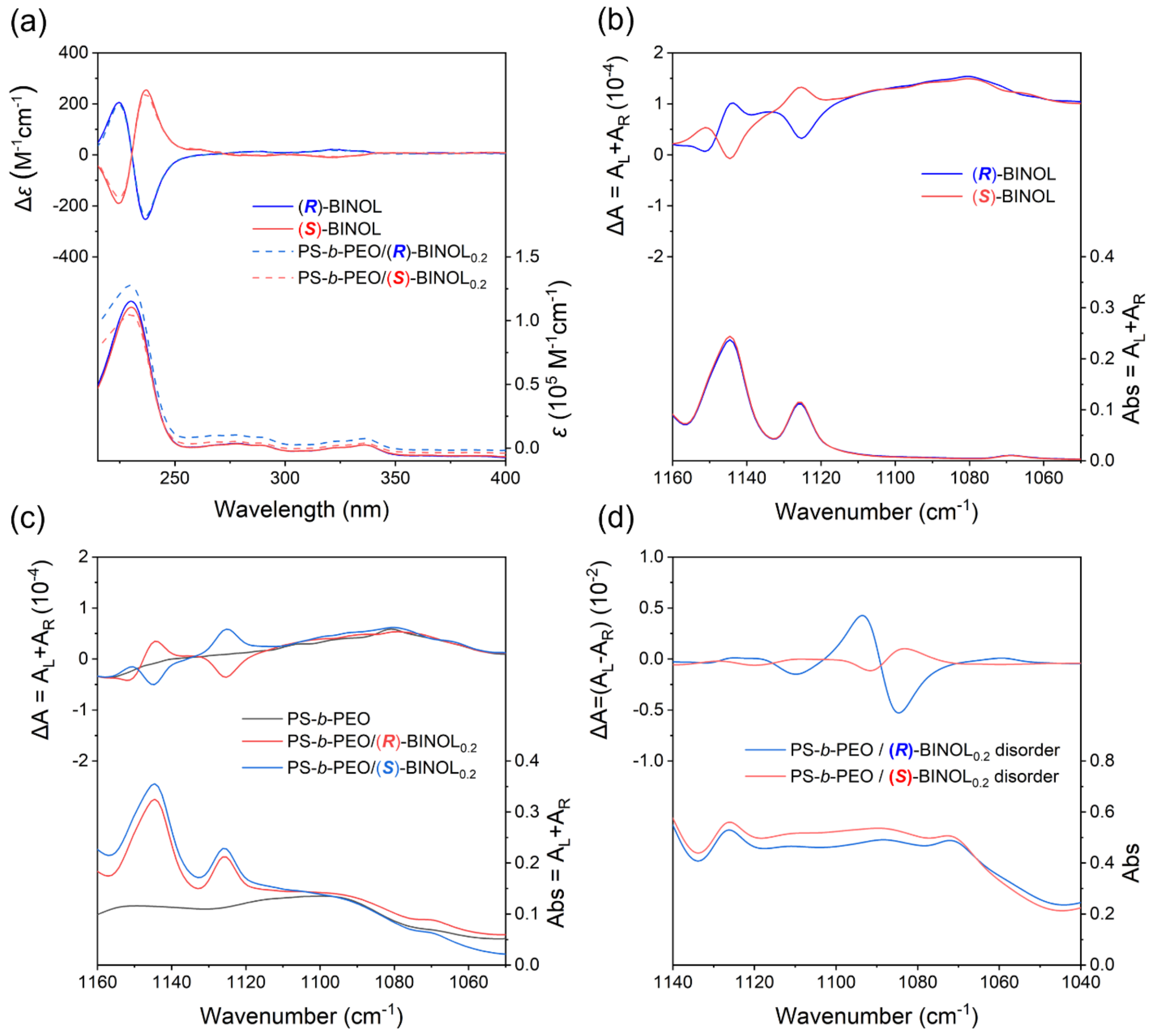

Figure S2. (A) ECD and corresponding UV-Vis spectra of (*R*)- and (*S*)-BINOL, along with PS-*b*-PEO/(*R*)-BINOL$_{0.2}$ and PS-*b*-PEO/(*S*)-BINOL$_{0.2}$ in solution. (B) VCD and corresponding FTIR spectra of (*R*)- and (*S*)-BINOL in solution. (C) VCD and corresponding FTIR spectra of PS-*b*-PEO, PS-*b*-PEO/(*R*)-BINOL$_{0.2}$ and PS-*b*-PEO/(*S*)-BINOL$_{0.2}$. (D) VCD and corresponding FTIR spectra of disordered PS-*b*-PEO/(*R*)-BINOL$_{0.2}$ and PS-*b*-PEO/(*S*)-BINOL$_{0.2}$ in the thin-film state.

To examine the ICD behavior of the PEO block in the PS-*b*-PEO (achiral host) driven by the chiral BINOLs (chiral guest), the chiroptical response of BINOLs were also investigated *via* ECD. **Figure 3(a)** shows the ECD spectra of (*R*)- and -(*S*)-BINOL in solution with characteristic multiple bisignate Cotton-bands, originating from π-π* transition of ^1B_b couplings of axially twisted naphthalene dimers of the intrinsic chiral BINOLs. After the association, identical ECD signals suggest that the H-bonding association would not affect the axial chirality of BINOLs at the ground state.

Consistently, similar results can be also found in the solid-state ECD spectra for both disordered morphology and H* (**Figure S1**). Furthermore, the consistency observed in all the ECD spectra implies that whether in the disordered state or within the self-assembled samples, BINOLs maintain the same conformation.[35]

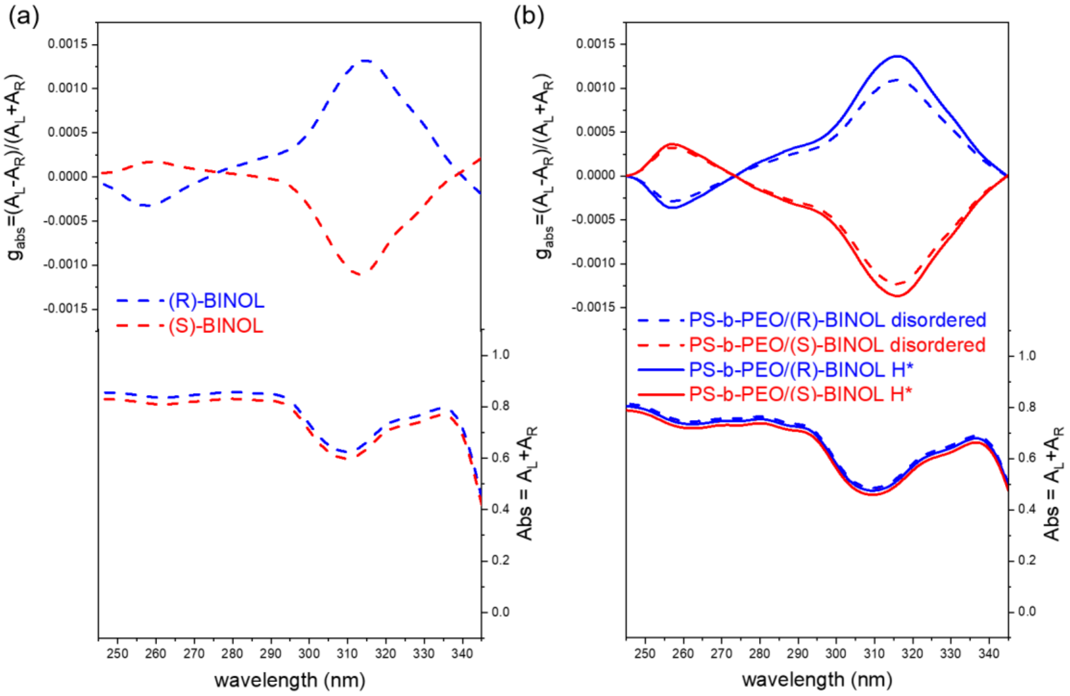

Figure S3. ECD and corresponding UV-Vis spectra of (a) (*R*)- or (*S*)- BINOL in the solid state and (b) those of PS-*b*-PEO/(*R*)-BINOL$_{0.2}$ and PS-*b*-PEO/(*S*)-BINOL$_{0.2}$ in the solid state with disordered and H* phases.

CPL activity of achiral PS-*b*-PEO associated with chiral BINOLs in solution and disordered phase

Figure S2(a) shows the CPL and corresponding PL spectra of intrinsic BINOLs, PS-*b*-PEO and PS-*b*-PEO/(*R*)- or (*S*)-BINOL$_{0.2}$ in a dilute solution, giving no significant variation on the chiroptic activities as compared to intrinsic BINOLs since there is no significant inter-chain chiral interaction from the PEO chains in solution even with association of the chiral BINOL. By contrast, a moderately enhanced CPL activity as compared to the intrinsic BINOL in the solid state (g_{lum} = 0.002) could be found in the mixture of PS-*b*-PEO/(*R*)- or (*S*)-BINOL$_{0.2}$ with the disordered phase (g_{lum} = 0.009) (**Figure S2b)**). The slightly amplified chiroptical activities suggest that randomly oriented static helical chains in the disordered state are not able to create significant amplification effect on aimed chiroptical activities. To further investigate the impact of hydrogen bonding between BINOL and PEO on the ICD an iCPL behaviors, we also introduced methoxy and dimethoxy derivatives of BINOLs, denoted as mBINOL and dmBINOL, respectively (**Figure S3**). This allowed us to systematically compare their effects with the previously studied BINOL. As shown in **Figure S4** and **S5**, the ECD and CPL patterns observed in solution for both mBINOL and dmBINOL remain consistent, whether blended with PS-b-PEO or employed independently, mirroring the behavior exhibited by BINOL. However, during the film preparation process, the dmBINOL sample fails to demonstrate amplified ICD (**Figure S6**), possibly attributed to the single OH group limited ability to establish a sufficiently strong hydrogen bond with PEO, exacerbated by steric hindrance introduced by the methoxy group. In the case of dmBINOL, it precipitates entirely(**Figure S7**), likely due to the absence of any hydrogen bonding interactions between PEO and dmBINOL.

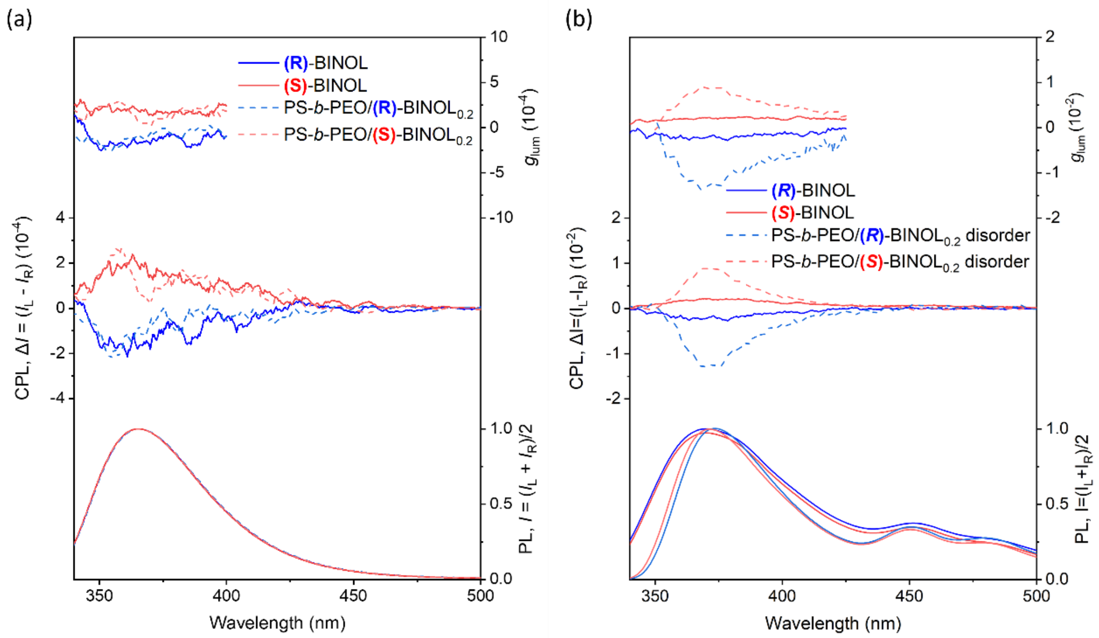

Figure S4 CPL and corresponding PL spectra of (a) (*R*)- and (*S*)-BINOL, PS-*b*-PEO/(*R*)-BINOL$_{0.2}$, and PS-*b*-PEO/(*S*)-BINOL$_{0.2}$ in THF solution and (b) those of (*R*)- and (*S*)- BINOL, PS-*b*-PEO/(*R*)-BINOL$_{0.2}$, and PS-*b*-PEO/(*S*)-BINOL$_{0.2}$ in the solid state with disordered morphology.

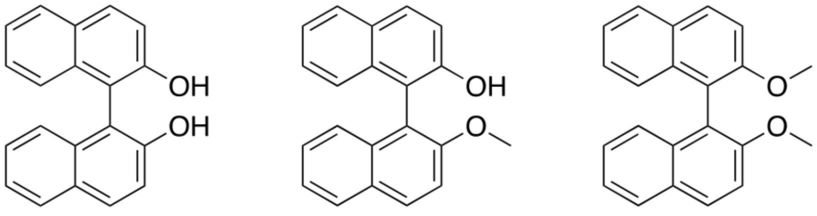

BINOL mBINOL dmBINOL

Figure S5 Chemical formula for BINOL and its methoxy derivatives dmBINOL and dmBINOL.

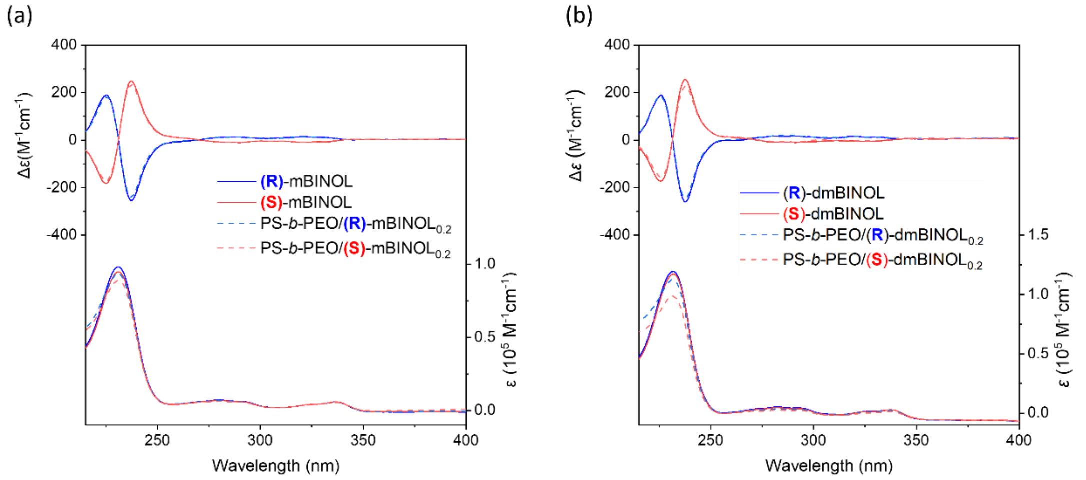

Figure S6 ECD and corresponding UV-Vis spectra of (a) (*R*)- and (*S*)-mBINOL, PS-*b*-PEO/(*R*)-mBINOL$_{0.2}$, and PS-*b*-PEO/(*S*)-mBINOL$_{0.2}$ and (b) those of (*R*)- and (*S*)-dmBINOL, PS-*b*-PEO/(*R*)-dmBINOL$_{0.2}$, and PS-*b*-PEO/(*S*)-dmBINOL$_{0.2}$ in THF solution at 22°C.

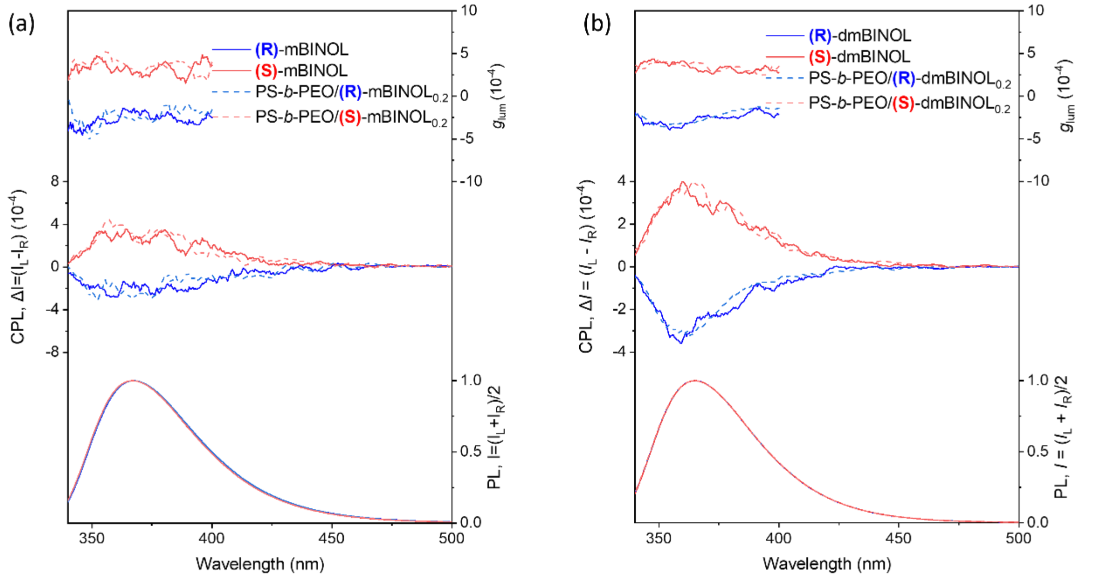

Figure S7 CPL and corresponding PL spectra of (a) (*R*)- and (*S*)-mBINOL, PS-*b*-PEO/(*R*)-mBINOL$_{0.2}$, and PS-*b*-PEO/(*S*)-mBINOL$_{0.2}$ and (b) those of (*R*)- and (*S*)-dmBINOL, PS-*b*-PEO/(*R*)-dmBINOL$_{0.2}$, and PS-*b*-PEO/(*S*)-dmBINOL$_{0.2}$ in THF solution.

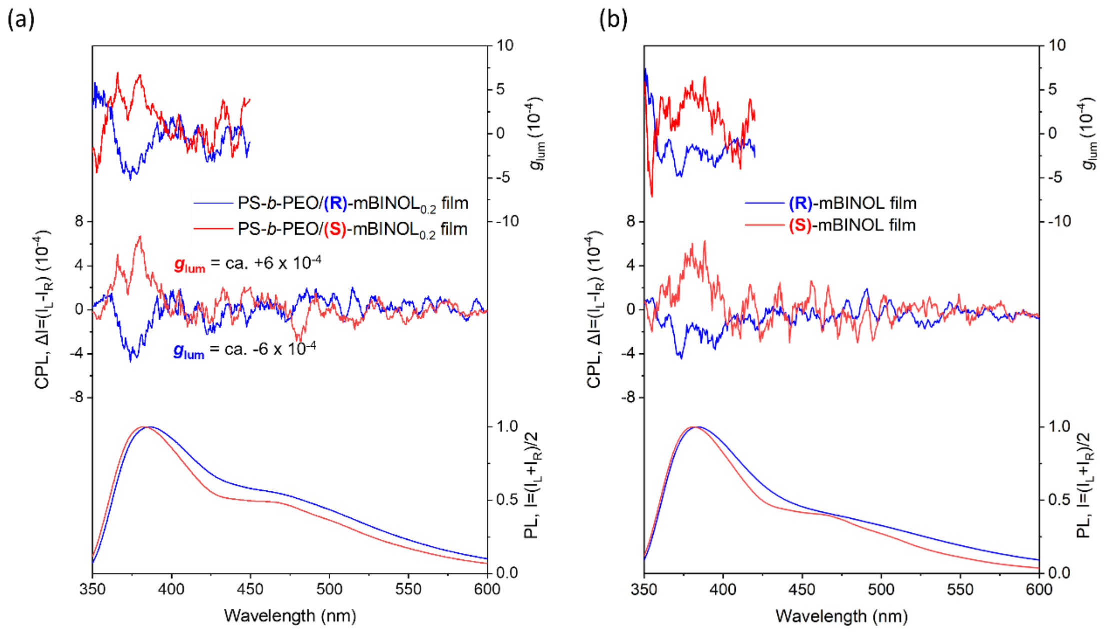

Figure S8 CPL and corresponding PL spectra of (a) S-*b*-PEO/(*R*)-mBINOL$_{0.2}$ and PS-*b*-PEO/(*S*)-mBINOL$_{0.2}$ in the solid state; (b) (*R*)-mBINOL and (*S*)-mBINOL in the solid state.

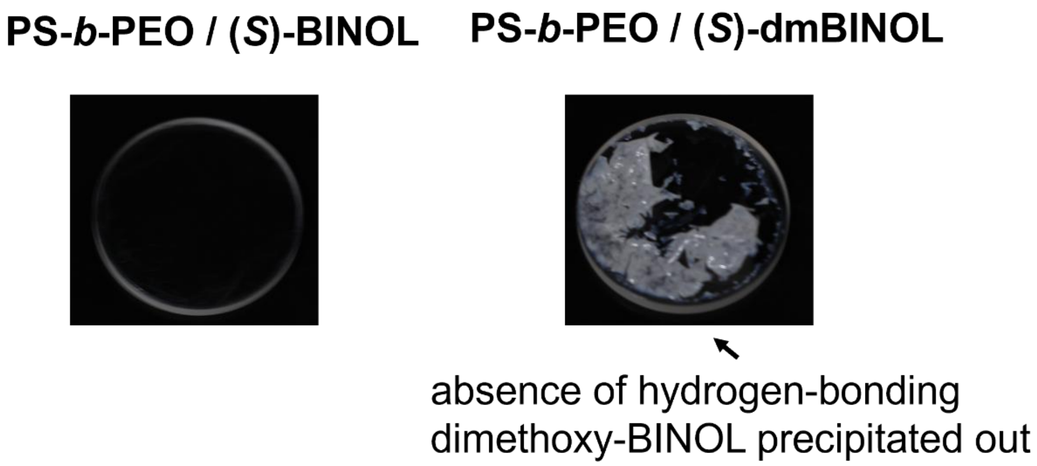

Figure S9 Photographs of the PS-*b*-PEO/(*S*)-BINOL$_{0.2}$ film and PS-b-PEO/(*S*)-dmBINOL$_{0.2}$ film on the quartz.

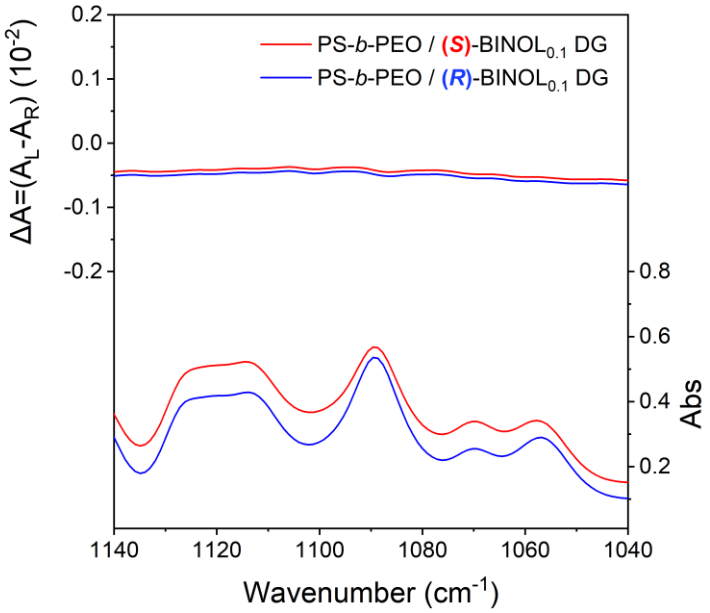

Figure S10 VCD and corresponding FTIR spectra of self-assembled DG of PS-*b*-PEO/(*R*)-BINOL$_{0.1}$ and PS-*b*-PEO/(*S*)-BINOL$_{0.1}$.

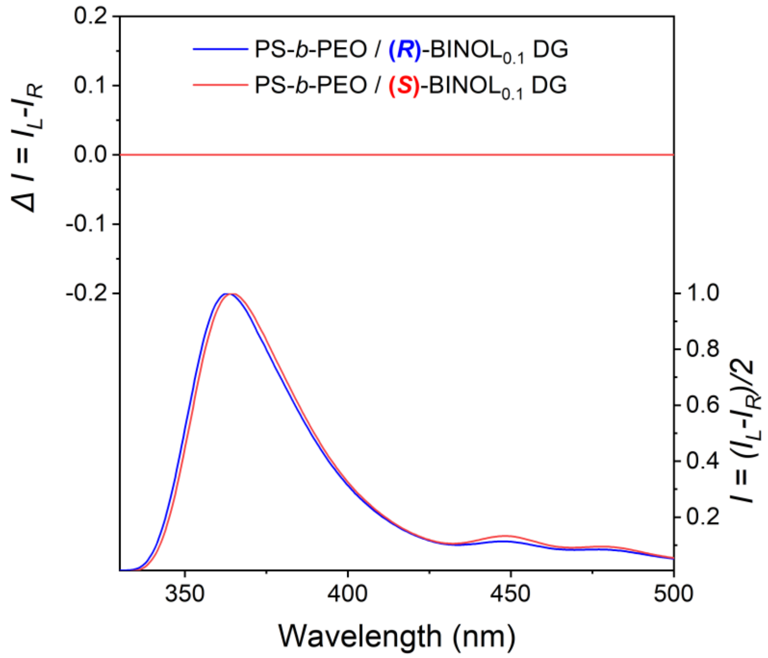

Figure S11 CPL and corresponding PL spectra of self-assembled DG of PS-*b*-PEO/(*R*)-BINOL$_{0.1}$ and PS-*b*-PEO/(*S*)-BINOL$_{0.1}$ in the solid state. For comparison, those of PS-*b*-PEO/(*R*)-BINOL$_{0.2}$ and PS-*b*-PEO/(*S*)-BINOL$_{0.2}$ with H* phase in the solid state are also shown.

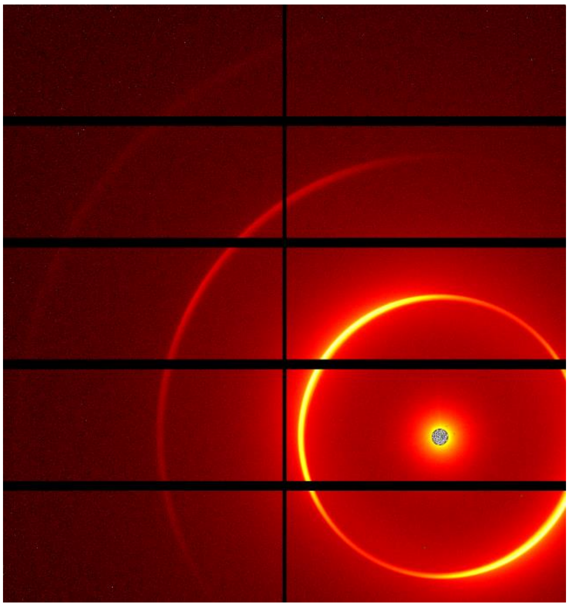

Figure S12 2D SAXS image of PS-*b*-PEO/(S)-BINOL$_{0.2}$

References

(35) Zhang, H.; Zheng, X.; Kwok, R. T.; Wang, J.; Leung, N. L.; Shi, L.; Sun, J. Z.; Tang, Z.; Lam, J. W.; Qin, A. In situ monitoring of molecular aggregation using circular dichroism. *Nat. Commun.* **2018**, *9* (1), 4961.
